# Toll-like receptor 9 negatively related to clinical outcome of AML patients

**DOI:** 10.1186/s43046-020-00027-3

**Published:** 2020-03-30

**Authors:** Waiel M. A. Al-Kahiry, Enas A. M. Dammag, Hadeel S. T. Abdelsalam, Hayat K. Fadlallah, Mona S. Owais

**Affiliations:** 1 Faculty of Medicine, Aden University, Aden, Yemen; 2 Faculty of Medicine, Taiz University, Taiz, Yemen; 3 Medical Laboratory Technology Department, Faculty of Allied Medical Sciences, Pharos University, Alexandria, Egypt; 4 Department of Hematology, Medical Research Institute, Alexandria University, Alexandria, Egypt; 5 Department of Clinical Pathology, Alexandria University Hospital, Fellow of Clinical Pathology, Alexandria Main University Hospital, Alexandria, Egypt

**Keywords:** TLR9, Acute myeloid leukemia, Outcome, Survival

## Abstract

**Background:**

Acute myeloid leukemia (AML) can modulate toll-like receptor-9 (TLR9) expression and activation. This study was conducted to elucidate the expression of TLR9 in AML patients and its relation to the prognosis of the disease.

**Results:**

The study included 40 newly diagnosed AML patients managed in the hospital in addition to 20 sex and age matched normal volunteers as control. TLR9 expression assay was conducted on peripheral blood samples of AML cases before the start of treatment as well as the controls by immunophenotyping. TLR9 expression was ranging from 0.10 to 2.40% in AML patients with higher expression among the control, ranging from 0.94 to 8.25%. The median TLR9 expression in AML patients was significantly lower with advanced cytogenetic risk score. It is not significantly differing in relation to patients’ sex, age group, and FAB type of AML. However, significant lower median expression was found in relation to clinical outcome. TLR9 expression ≤ 1% showed lower median overall survival time when compared to those with > 1% expression.

**Conclusion:**

This study concluded that AML patients express TLR9 in leukemic cells with very low percentage. This expression was negatively related to the clinical outcome.

## Background

Acute myeloid leukemia (AML) is a clonal aggressive stem cell disorder characterized by overproliferation and failure of differentiation of stem cells leading to loss of normal hematopoietic function and accumulation of non-functional myeloid cells (myeloblasts) [1]. Recently, several defects in T cell function, including proliferation and cytokine production, have been proposed to be associated with the pathogenesis of AML [2].

Studies reported that the Bruton’s tyrosine kinase (BTK) is expressed in about 80% of human AML patients. It promotes AML cell proliferation and survival via activation of several signaling pathways that involve signaling from toll-like receptor-9 (TLR9) and FLT3-ITD, which trigger various context-dependent downstream transcriptional programs [3, 4].

Toll-like receptor-9 is a protein encoded in human by the *TLR9* gene which is located at chromosome 3p21.2. It is a member of the TLRs group known previously as CD289 [5]. TLR9 is an intracellular receptor expressed in immune system cells such as macrophages, dendritic cells, natural killer cells, and other antigen presenting cells. TLR9 binds the DNA of viruses and bacteria. It can trigger signaling cascades for a pro-inflammatory cytokine response [6, 7]. Cancer and tissue damage can modulate TLR9 expression and activation [8, 9].

TLR9 expression promotes an inflammatory process by inducting aberrant level of cytokines as well as inhibiting cell cycle induced apoptosis. In a study of cancer cells from 133 prostate cancer patients, higher TLR9 expression was found to be accompanied with aberrant level of cytokines, inhibiting cell cycle induced apoptosis [10].

As the TLR9 plays a pivotal role in triggering the immune response and the inflammatory process, then its altered expression had been suggested in various malignancies. Therefore, it is important to investigate the TLR9 expression in AML patients and to assess whether it is related to the prognosis of AML or not.

## Methods

### The aim, design, and setting of the study

This study aims to determine the TLR9 expression in AML patients and its relation to the prognosis of the disease.

This is a prospective study conducted in the Hematology Department of the hospital from Aug, 2016 to Aug, 2018.

### Participants and description of materials

Participants were 40 newly diagnosed AML patients managed in the hospital in addition to 20 sex and age matched normal volunteers as control.

The AML patients with mixed lineage expression, autoimmune diseases, coexisting secondary malignancy, hepatitis C viral infection, and bilharziasis were excluded. In patients presented with infection, the flow cytometry test for TLR9 was done after infection control by antimicrobial therapy for 7 to 10 days.

AML was diagnosed based on the finding of ≥ 20% blasts in the bone marrow, and the flow cytometry characterized the leukemic blasts using acute panel of monoclonal antibodies labeled by fluorescein isothiocyanate (FITC) (green) or phycoerythrin (PE) (red).

Cytogenetic studies were done by fluorescence in situ hybridization (FISH) analysis, and the patients were classified as favorable, intermediate, and poor risk groups based on the cytogenetic findings [11]. Favorable cytogenetic risk is defined by the presence of t(8;21)(q22;q22) and/or detection of an AML1-ETO translocation product, independent from additional cytogenetic findings. Or the presence of an inv(16)(p13p22) or a t(16;16)(p13;p22) and/or detection of a CBFβ-MYH11 translocation product, independent from additional cytogenetic findings. Poor cytogenetic risk is defined by the presence of del(5q) or monosomy 5, del(7q) or monosomy 7, inv(3)(q21q26.2) or t(3;3)(q21;q26.2), 11q23 aberrations, or complex-aberrant karyotype comprising of three or more aberrations not involving low-risk aberrations. Intermediate cytogenetic risk is defined by the presence of all other aberrations not included in low or high risk [11].

### The assay of TLR9 expression

The assay of TLR9 expression was performed on peripheral blood samples of AML cases before the start of treatment as well as the healthy controls. TLR9 is an intracytoplasmic antigen, detected after permeabilization using Tween 20 and paraformaldehyde for gentle fixation of cells. The monoclonal antibodies (MoAbs) of Abcam, Anti-TLR-9 antibody [5G5] (FITC) ab58864, the United Kingdom was used in analysis of washed cells by flow cytometer.

**Fig. 1:**
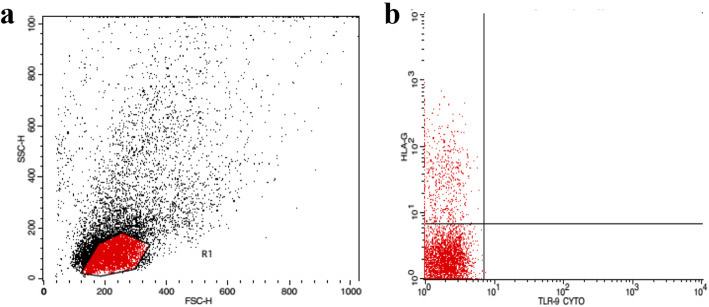
Flow cytometry of one of the cases. **a** Gating of lymphocytes. **b** Expression of TLR-9

**Fig. 2:**
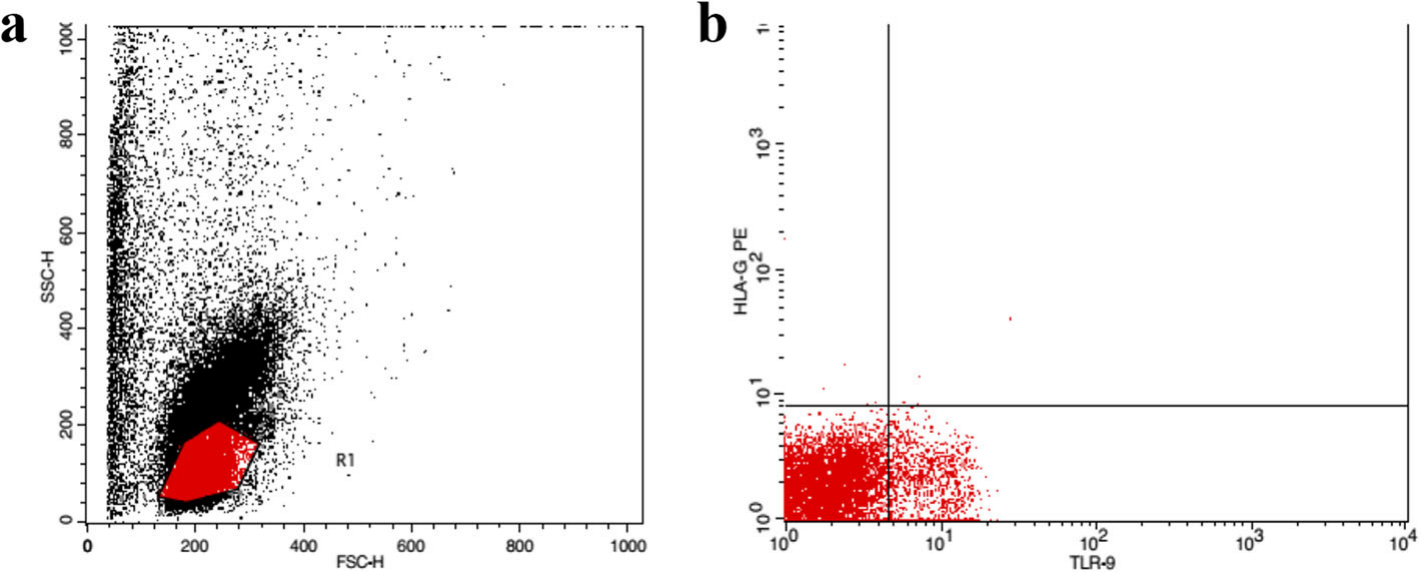
Flow cytometry of one of the controls. **a** Gating of lymphocytes. **b** Expression of TLR-9

Immunofluorescence on the viable leukemic cells in suspension was analyzed using Becton Dickinson, FACS caliber flow cytometer equipped with the Cell Quest software [USA]. Gating was done around the leukemic blasts on the forward versus side scatter plots in the initial diagnostic specimens, while in the control specimens gating was done around the mononuclear cells on the forward versus side scatter plots. Representative histograms for gating and expression in one of the cases and one of the controls as follows (Fig. 1, 2).

### Clinical outcomes and survival analysis

Clinical outcomes were based on the response to treatment. The response to treatment was defined according to the 2017 European Leukemia Network (ELN) recommendations [12], with evidence of persistent leukemia by blood and/or bone marrow examination.

Complete remission (CR) was defined by bone marrow blasts < 5% durable for at least 28 days, absence of blasts with Auer rods, absence of extramedullary disease, absolute neutrophil count > 1.0 × 10^9^/L, platelet count > 100 × 10^9^/L, and independence of red cell transfusions. Partial remission (PR) was defined by all hematologic criteria of CR, decreased bone marrow blast percentage from 5 to 25% and decreased pretreatment bone marrow blast percentage by at least 50%. Refractory disease (RD) was defined as the failure to achieve CR or PR, only includes patients surviving ≥ 7 days following completion of initial treatment, with evidence of persistent leukemia by blood and/or bone marrow examination.

The follow-up period was 24 months, from Aug, 2016 to Aug, 2018. Survival analysis was described as the overall survival time.

This study was ethically approved by the ethical committee of the hospital. Patients and their relatives were informed about the objective of the study and their written consents were taken prior to inclusion to this study.

### Statistical analysis

Data processing was performed by the SPSS v.24 which revealed non-parametric distributed data a part of age and hemoglobin level of the studied groups. For these, parametric *t* tests were used. For other parameters, non-parametric tests were conducted (Mann-Whitney *U* test for two medians and Kruskal-Wallis test for more than 2 medians). Qualitative variables were tested by the chi-square test. The ROC curve for TLR9 was conducted to estimate the cutoff value with the highest sensitivity. The Kaplan Meier method was used to describe the median overall survival time in regard to the cutoff value of TLR9 and tested by the log-rank test. All tests were conducted with the 95% confidence interval and *p* values of ≤ 0.05 were considered statistically significant.

## Results

In this study, the percentage of male patients was higher than female (60% vs. 40%). The male to female ratio was (1.5:1). The mean age was 50.85 ± 17.9 years with higher frequency of patients younger than 65 years of age. TLR9 expression was ranging from 0.10 to 2.40% in AML patients. Higher expression was recognized among the control, ranging from 0.94 to 8.25%. The median TLR9 expression was significantly lower among AML patients when compared to the control (1.30% vs. 4.10%) (Table 1).

**Table 1:** Sex, age, TLR9 expression, and some laboratory parameters of the studied patients with acute myeloid leukemia compared to the control

Item		AML patients (***n*** = 40)	Control (***n*** = 20)	***p*** value
Sex	Male	24 (60.0%)	12 (60.0%)	1.0
	Female	16 (40.0%)	8 (40.0%)	
Age (years)	< 65	26 (65.0%)	14 (70.0%)	0.699
	≥ 65	14 (35.0%)	6 (30.0%)	
	Mean ± SD	50.85 ± 17.9	48.2 ± 14.8	0.570
TLR9 (%)	Median (Min.-Max.)	1.30 (0.10-2.40)	4.10 (0.94-8.25)	0.001*
Hemoglobin concentration (g/dl)	Mean ± SD	7.61 ± 2.4	14.6 ± 2.4	0.0001*
Total leukocytic count (X10^9^/L)	Median (Min.-Max.)	18.5 1.2-235.0	7.3 4.1-10.0	0.0001*
Platelets count (X10^9^/L)	Median (Min.-Max.)	42.0 14.0-140.0	231.0 180-430	0.0001*
Blasts (%)	Median (Min.-Max.)	54.0 25.0-92.0	--	--

*p value < 0.05 is statistically significant

*TLR* toll-like receptor

The median of hematological parameters in the studied patients showed marked anemia, leucocytosis, and thrombocytopenia. The bone marrow blasts count ranges from 25 to 92% with a median of 54% (Table 1).

**Fig. 3:**
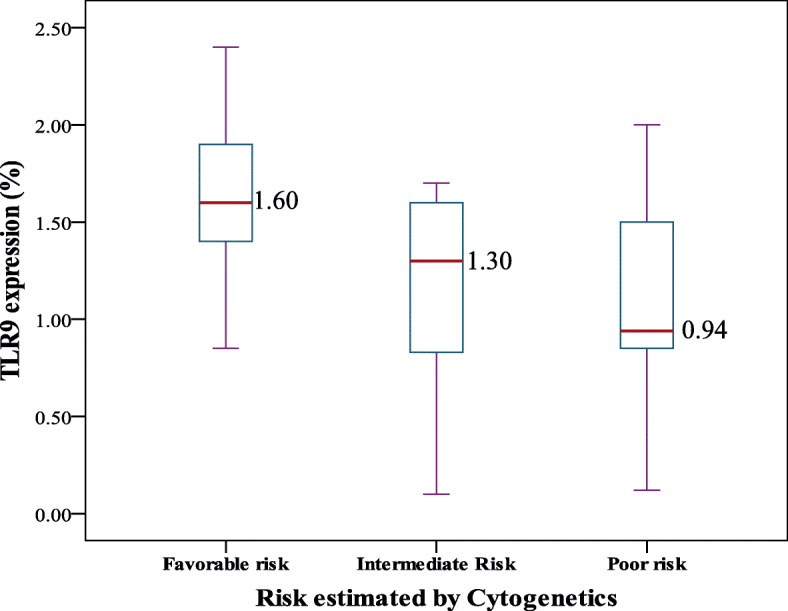
The median TLR9 in regard to the risk estimated by cytogenetic analysis of the studied AML patients

The median TLR9 expression in the studied AML patients was significantly lower with advanced risk score estimated by cytogenetic findings (*p* = 0.027) (Fig. 3).

This expression did not show any significant difference in relation to patients’ sex, age, group, and FAB type of the acute myeloid leukemia. However, significant lower median expression was found in relation to clinical outcome (Table 2).

**Table 2:** The median of TLR9 expression in relation to some parameters in AML patients

Parameter		№	TLR9 (%)		***p***-value
			Min.-Max.	Median	
Sex:	Female	16	0.10-2.40	1.35	0.699
	Male	24	0.12-2.40	1.30	
Age group	< 65 years	26	0.12-2.40	1.35	0.150
	≥ 65 years	14	0.10-2.00	1.07	
FAB type	AML –M1	1	1.20	1.20	0.487
	AML –M2	13	0.12-2.00	1.40	
	AML –M3	7	0.90-2.40	1.60	
	AML –M4	12	0.10-2.00	1.30	
	AML –M5	5	0.12-2.30	1.30	
	AML –M6	1	1.60	1.60	
	AML –M7	1	0.38	0.38	
Outcome	Complete remission	8	0.90-2.40	1.85	0.006^*^
	Partial remission	12	0.12-2.30	1.50	
	Refractory	4	0.12-1.60	0.70	
	Died	14	0.10-1.60	0.925	
	Lost during follow up	2	1.30-1.60	1.45	

^*^Statistically significant

**Fig. 4:**
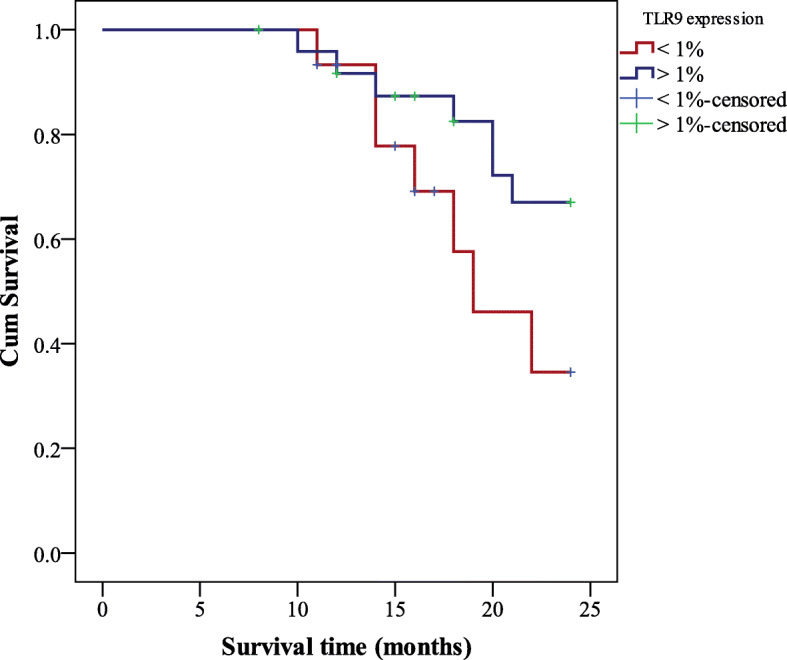
The median overall survival time in regard to the cutoff value of TLR9

The ROC curve for TLR9 expression was drawn and the cutoff value with the highest sensitivity was selected (TLR9 expression of 1% with sensitivity, 0.923 and 1- specificity, 0.929). With this cutoff value, patients with TLR9 expression ≤ 1% showed lower median overall survival time when compared to those with > 1% expression (19.3 months vs. 21.6 months). However, this difference was not found statistically significantly (*p* = 0.087) (Fig. 4).

## Discussion

TLR9 is a nucleotide sensing TLR, localized to the endosomal/lysosomal compartment. Activated by unmethylated cytidine-phosphate-guanosine (CpG) dinucleotides. It acts via MYD88 and TRAF6, leading to NF-kB activation, cytokine secretion, and the inflammatory response [13, 14].

Gupta et al [15] reported that TLR9 triggering/STAT3 inhibition to reprogram leukemic cells into antigen-presenting cells and trigger T cell responses. TLR9 expression in this study showed significantly very low percentage of expression when compared to the control. In the study of Takeshita et al. [13], they found that the expression of TLR9 is a prerequisite for activation of human cell by CpG DNA. They observed that when the levels of TLR9 mRNA increase, the responsiveness of human cells to CpG DNA increases. This finding supports the hypothesis of immune escape by leukemic cells because leukemia is characterized by an impaired immune system. This may be achieved in part by dysregulation of TLRs. It is possible that in AML, the leukemia cells downregulate the expression of TLRs to guarantee their survival.

In this study, the TLR9 was ranging from 0.10 to 2.40% with a median of 1.30%. This finding coinciding with that reported by Morsi et al [16], where they found a range of (0.941-1.571%) with a mean of 1.35% among 15 AML patients.

The aim of the current study was to evaluate the TLR9 expression at diagnosis of AML as an additional prognostic factor for prediction of clinical outcome and survival of patients. It was found that the initial expression of TLR9 in AML patients was significantly lower in AML patients with intermediate and poor risk cytogenetic scores as well as in AML patients who died or considered clinically as refractory to standard therapy regimens.

Rybka J et al. [17] studied the mRNA expression of TLR9 among 103 AML patients before and after induction therapy. However, they did not find a significant difference in patients with complete remission after the first cycle of chemotherapy when compared to patients who failed to respond to treatment. The study of Rybka J et al [17] did not extend beyond the induction phase.

The study of Elmaagacli A et al. [18] reported that TLR9 influence the outcome in AML patients transplanted from HLA identical sibling donors.

The survival of AML patients was generally poor. When we followed the studied patients for 24 months, it was found that patients with lower TLR9 expression (≤ 1%) showed lower median overall survival time when compared to those with expression of > 1%.

We did not find any study for the survival of AML patients in relation to TLR9 expression before the start of chemotherapy. The current study is considered the first study to deal with survival of AML patients in relation to TLR9 expression before the start of chemotherapy.

There are scarce studies that compared the survival in relation to TLR9 expression among other malignancies. Belmont L et al [19] studied tumor biopsies of 61 patients with early stage non-small cell lung cancer (NSCLC) and mouse model with induced lung cancer. They reported that TLR9 expressed by mononuclear cells was associated with a worse survival in the mouse model of lung adenocarcinoma and in patients with early-stage NSCLC.

In 2006, El-Andaloussi A et al [20] reported that stimulation of TLR9 with CpG oligodeoxynucleotides enhances apoptosis of glial cells of glioma and prolongs the survival of mice with experimental brain tumors. In 2008, Meng Y et al. [21] studied 37 histologically confirmed patients with glioblastoma and one primary central nervous system lymphoma. They did not report any significant relationship between TLR9 expression and survival in the studied patients.

The findings of the current study suggest that at presentation of AML patients, leukemic cells hide the TLR9 expression to escape immune surveillance. The more the ability to lurk the receptors, the worse the clinical outcome.

In the study of Krysko et al [22], they showed that TLR9 has been described as an apoptosis sensor by recognizing damage-associated molecular patterns (DAMPs) which may contribute to drug resistance in AML patients, where a high turnover of blasts in the bone marrow, especially under the influence of cytotoxic drugs, might stimulate the identified TLR9/BTK transducer module via DAMP release.

AML is a common form of acute leukemia that is still associated with poor survival of patients, mainly those who failed to respond to standard therapy. These patients are in immense need for new targeted therapies for better cure rate and longer survival among AML patients [23]. The current study found that TLR9 may be one of the receptors that should be studied and targeted aiming to obtain higher rate of complete cure and survival for the benefit of AML patients.

Krieg A [24], in his review about the therapeutic potential of TLR9 activation, reported that the targeted activation of TLR9 by using CpG oligodeoxynucleotide may enhance the treatment of cancer. He reported also that TLR9 could be a target for which both agonists and antagonists could find therapeutic application on different clinical settings.

## Study limitation

The study limitations included a small sample size, as well as heterogeneity of the included AML cases due to different FAB types.

## Conclusion

This study concluded that at presentation of AML patients, they express TLR9 with very low percentage. The TLR9 expression was negatively related to the clinical outcome, but not significantly related to the overall survival of AML patients. This study recommended the use of TLR9 agonists in poor risk groups AML patients aiming to help changing their outcome.

## Data Availability

The datasets used and/or analyzed during the current study are available from the corresponding author on reasonable request.

## Abbreviations

AML: Acute myeloid leukemia
BTK: Bruton’s tyrosine kinase
CpG: Cytidine-phosphate-guanosine
CR: Complete remission
DAMPs: Damage-associated molecular patterns
DNA: Deoxyribonucleic acid
ELN: European Leukemia Network
FAB: French American British
FISH: Fluorescence in situ hybridization
FITC: Fluorescein isothiocyanate
FLT3-ITD: FMS like tyrosine kinase 3 - internal tandem duplication
MoAbs: Monoclonal antibodies
mRNA: Messenger ribonucleic acid
NSCLC: Non-small cell lung cancer
PE: Phycoerythrin
PR: Partial remission
RD: Refractory disease
ROC: Receiver operating characteristic
SPSS: Statistical Package for Social Sciences
STAT3: Signal transducer and activator of transcription 3 (gene)
TLR9: Toll-like receptor-9
